# Effect of short-term nutritional supplementation of green microalgae on some reproductive indicators of Anglo-Nubian crossbred goats

**DOI:** 10.14202/vetworld.2023.464-473

**Published:** 2023-03-17

**Authors:** Maria Raquel Lopes Silva, Juliana Paula Martins Alves, César Carneiro Linhares Fernandes, Camila Muniz Cavalcanti, Alfredo José Herrera Conde, Alessandra Façanha Bezerra, Anne Caroline Santos Soares, Dárcio Ítalo Alves Teixeira, Anibal Coutinho do Rego, Davide Rondina

**Affiliations:** 1 School of Veterinary Medicine, Ceará State University (UECE), Fortaleza, Ceará, 60714-903, Brazil; 2 School of Veterinary Medicine, University of Fortaleza (UNIFOR), Fortaleza, Ceará, 60811-905, Brazil; 3 Department of Animal Science, Federal University of Ceará (UFC), Fortaleza, Ceará, 60021-970 Brazil

**Keywords:** Doppler, follicles, goat, microalga, ovarian blood flow, ovarian response

## Abstract

**Background and Aim:**

Despite the wide spectrum of uses, one of the chief drawbacks to expanding microalgae as a food supplement in livestock is the lack of a regimen protocol with established dosage and time length of supplementation. Therefore, this study aimed to investigate the effect of short-term supplementation with increasing doses of microalgae on ovarian response in goats reared in northeast Brazil.

**Materials and Methods:**

Twenty-eight goats had their follicular waves synchronized using three injections of a prostaglandin analog at 7-day intervals. Goats were allocated to groups that received daily oral Chlorella supplementation for 7 days, respectively: 5 g, GMA5 group (n = 7), 10 g (GMA10; n = 7), and 20 g (GMA20; n = 7). The control group (GMA 0; n = 7) received a drench of water.

**Results:**

The groups showed a quadratic increase (p = 0.0156) in kidney fat thickness but there was a significant reduction in dry matter intake in the GMA20 group. The GMA20 group showed higher glucose levels and glutathione peroxidase (p < 0.05). There was a decrease in plasma cholesterol (p < 0.05) in the 10 and 20 g treatments. The number of total follicles increased quadratically. Follicles <3 mm increased linearly (p = 0.0113) for microalgal supply. The GMA10 and GMA20 groups had the highest values (p < 0.05) among the treatments. After inducing ovulation, there was a significant increase in follicles >3 mm in the GMA10 group, which also showed a greater (p < 0.05) area of intraovarian blood perfusion and pulsatility index of the ovarian artery.

**Conclusion:**

We conclude that for 7 days of supplementation, the administration of 10 g of microalgae appears to be the most efficient dosage for stimulating the ovarian response in tropical goats.

## Introduction

Microalgae are unicellular with similar metabolic characteristics, among which green algae such as *Chlorella* belong to the chlorophyte family and present a different species [1]. At present, there is growing interest worldwide in the use of microalgae as a source of biomass for the production of biofuels and use in wastewater treatment and value-added materials for the biopharmaceutical and nutraceutical industries [2]. In 2019, the global production of microalgae and algae was 35.8 million tons [3]. Among the primary producers of microalgae biomass are China, Japan, Taiwan, and the United States [4]. Nutritionally, microalgae biomass offers several components such as dietary fiber, polyphenols, carotenoids, phycobiliproteins, polysaccharides, vitamins, sterols, and polyunsaturated fatty acids (PUFAs), including ω-3 eicosapentaenoic acid (20:5 n-3) and docosahexaenoic acid (DHA, 22:6 n-3) [5, 6].

Different bioactive compounds are beneficial to human nutrition with therapeutic potentials, such as antioxidant and anti-inflammatory actions [7], antitumor effects [8], and insulin control and resistance [9]. In ruminants, the authors described several types of supplementation regimens with microalgae or marine algae. Their results indicated the beneficial action of PUFAs on the quality of milk [10–12] and meat [13, 14]. In tropical regions, the value of microalgae as a supplement in animal nutrition is widely recognized as a growth promoter [15] and a milk quality improver [16]. However, in goats, the available data are still limited and contradictory [17] and successively [18] reported significant changes in milk quality supplemented with 10 g/day of *Chlorella* for 43 and 21 days, respectively. In contrast, Moreno-Indias *et al*. [19] and Tsiplakou *et al*. [20] found no effects on milk composition when 11 g and 5 g/day of *Chlorella* were supplied for 28 and 40 days, respectively. Despite the wide spectrum of uses, one of the chief drawbacks to expanding microalgae as a food supplement in livestock is the lack of a regimen protocol with established dosage and time length of supplementation. This is evident when we analyze information on the reproductive response, especially in goats, considering the scarcity of contributions in this area. The available data suggest that dietary supplementation of microalgae can induce follicular growth in gilts [21] and goats [22], but it does not provide information about which dosage is more effective for the ovary. However, follicular and ovulatory responses are subordinated to the duration of dietary supplementation and supply levels [23]. Nutritional balance is essential for improving reproductive efficiency [24]. Changes in nutrient intake before mating can significantly alter the ovarian response and embryo quality at the implantation stage [25]. However, one of the obstacles in many tropical areas, such as northeast Brazil, in the creation of sheep and goats remains the maintenance of nutritional status during prolonged seasonal food shortages. In these areas, the use of reproductive technologies is subordinate to the availability of high-quality foods that are often competitive with the human diet [26].

Therefore, this study aimed to determine whether increasing doses of green microalgae (*Chlorella pyrenoidosa)* for short periods influenced glucose and cholesterol concentrations, oxidative stress, ovarian-intraovarian blood flow, follicle development, and ovulatory rate in goats reared in northeast Brazil.

## Materials and Methods

### Ethical approval

The study followed the ARRIVE 2.0 guidelines [27]. The study was approved by the Ethics Committee on Animal Experimentation of the Ceará State University (Approval number: 04029516/2021). The sample collection was performed according to standard sample collection procedure, without any harm to the animals.

### Study period and location

This study was conducted in May and June of 2021 at the facilities of the laboratory of nutrition and ruminant production (LANUPRUMI) of the “Esaú Accioly Vasconcelos” experimental farm School of Veterinary Medicine, Ceará State University, located in Guaiuba, Ceará, in the equatorial zone (4°2′23″ S and 38°38′14″ W), Brazil. This area is characterized by a constant photoperiod regimen, has a warm, tropical, sub-humid climate with a mean annual rainfall and temperature of 904.5 mm and 26–28°C, respectively, with two distinct seasons: rainy, from February to May, and dry, from June to January.

### Animals and experimental design

The animals used in this experiment were 28 Anglo-Nubian crossbred with indigenous goats adult and pluriparous goats that were homogeneously distributed within treatments (p > 0.05) considering age (44.1 ± 8.9 months; overall mean ± Standard deviation), body weight (BW) (37.3 ± 3.8 kg), and body condition score (2.8 ± 0.1, from 1 to 5 score).

**Figure-1:**
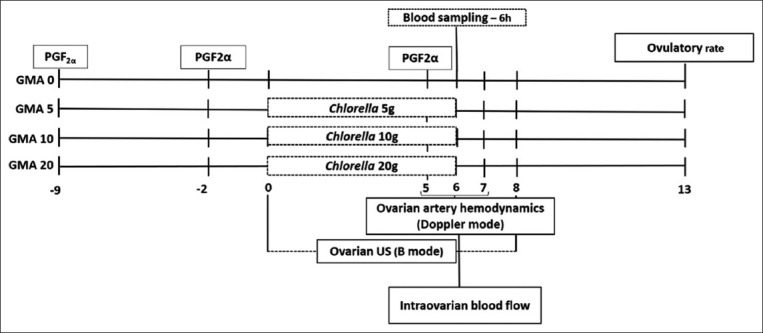
General experimental design, including dietary treatments and hormonal protocol.

All animals received the same diet during the 15 days of acclimation based on Bermuda grass hay and concentrate. The diet was provided to satisfy the nutritional requirements of adult non-dairy goats [28] for breeding. The experimental animals were kept in collective stalls and divided according to feeding group, with free access to mineral supplements and water. The diet was provided twice daily (07:00 and 15:00). Orts were collected daily and weighed weekly to determine the intake and extent of acceptance of the dietary supplements by the animals. All goats had synchronized estrus and follicular waves as described by Viñoles *et al*. [29] by administering three injections of 100 mg of the prostaglandin F2 alpha (PGF2α) analog D-cloprostenol (Prolise-Tecnopec, São Paulo, Brazil), at 7 days intervals (Figure-1). The third application of the PGF2α analog was aimed at promoting estrus and ovulation simultaneously in all goats.

Three groups were orally administered increased doses of green microalga (*C. pyrenoidosa* spp.; Sunrise Nutrachem Group Co., Ltd, Hongkong Middle Road, Qingdao, China, Figure-1): 5 g (group GMA5; n = 7), 10 g (group GMA10; n = 7), and 20 g (group GMA20; n = 7). Oral supplementation was based on a solution of dry microalgae powder with 80 mL of water provided twice daily (07:00 and 15:00 h) before feeding. The control group (GMA0; n = 7) received a drench of 80 mL of water for 7 days. The baseline diet was maintained as the control group.

During the experimental period, air temperature and humidity were recorded using a digital thermo-hygrometer (AK624, AKSO, RS, Brazil). The data are provided with an accuracy of 0.1°C. All parameters were measured in the pens.

### Diets and chemical composition

The feed ingredients and average chemical composition of the diet are presented in Table-1. The proximate composition of the diet samples was determined according to AOAC [30]. Feed and ort samples were dried in a forced-air-circulation oven at 55°C for 72 h and ground in a Wiley mill to pass through a 1 mm screen. The samples were analyzed for dry matter (DM) (Method 934.01), ash (Method 942.05), ether extract (Method 920.39), and crude protein (Method 978.04) [30]. Neutral detergent fiber was determined using α-amylase without the addition of sodium sulfite (Method 973.18), following the recommendations of Goering and van Soest [31]. The acid detergent fiber content was determined using the method described by Goering and van Soest [30]. Non-fibrous carbohydrate values were calculated following [32].

**Table-1:** Proportion of ingredients and chemical composition of base diet and green microalgae (g/kg of DM).

Composition	Diet	Microalgae
Ingredient, g/kg of DM		
Bermudagrass hay	600	
Ground corn grain	240	
Soybean meal	12	
Wheat bran	128	
Mineral mixture^a^	20	
Chemical fraction		
Dry matter, g/kg as-fed basis	884	919
Crude protein, g/kg of DM	112	589
Ether extract, g/kg of DM	21	198
Ash, g/kg of DM	78	72
Neutral-detergent fiber, g/kg of DM	559	178
Acid-detergent fiber, g/kg of DM	270	5
Non-fibrous carbohydrates, g/kg of DM	325	155

a Caprine Premix Dourado^®^, Ceará, Brazil. Premix containing (per kg): Ca 475 g, *P* 35 g, K 12.5 g, Na 20 g, Mg 61.5 g, Zn 850 mg, Co 15 mg, Cu 200 mg, S 75 mg, F 125 mg, I 20 mg, Mn 700 mg, Se 7 mg, Vitamin A 7,500 UI, Vitamin D 15,000 UI, Vitamin E 570 UI, DM=Dry matter

For *C. pyrenoidosa*, total lipid extraction was performed using a modified version of Bligh and Dyer’s method [33], where the samples were mixed with methanol-chloroform-distilled water (2:1:0.8, v/v) and subjected to cell disruption using probe sonication at a resonance of 40 kHz and 80 W for 10 min and transferred into a funnel with a filter paper. The lipids were vacuum filtered. The lipid content was quantified gravimetrically.

### Goat *in vivo* performance traits

Adipose mass was verified by ultrasonography on the −2^th^ day and on the 14^nd^ of the experimental period (Figure-1) by measuring the subcutaneous loin fat thickness between the 3^rd^ and 4^th^ lumbar vertebrae, following the methodology of Teixeira *et al*. [34], and to estimate visceral fat, measured the thickness of kidney fat behind the 13^th^ rib, following the methodology of Härter *et al*. [35]. A convex transducer with a frequency of 3.5 MHz (model Z5 Vet; Mindray Bio-Medical Electronics Co., Shenzhen, China) was used for kidney imaging. Images were captured in triplicate and measured using the previously calibrated ImageJ program (ImageJ, National Institutes of Health, Millersville, USA). During the evaluation, the animal was kept stationary, the areas on the right side of the body were shaved, and the gel was used as a coupling agent to improve the quality of the images. Goats were weighed on the same dates.

### Ultrasonography analysis

#### Ovarian follicular dynamics and ovulation rate

Ultrasonography was performed once a day from the 0^th^ day to 7^th^ day (Figure-1). Ovarian images were obtained with B-mode ultrasound equipment using a 5 MHz linear transrectal probe (model Z5 Vet; Mindray Bio-Medical Electronics Co.), as described by Alves *et al*. [36]. For image capture and analysis, the Image J software (Image J, National Institutes of Health) was used, which was previously calibrated.

An ovarian follicular wave was defined as the emergence of a group of small follicles (<3 mm) that gave rise to one or more large follicles (≥3 mm). The day of wave emergence was considered the day when the largest follicle of that wave had reached 3 mm in diameter. The growth phase was defined as the period during which a large follicle grew from 3 mm to its maximum diameter. The regression phase was the period from maximum follicle diameter to a diameter of 3 mm.

The ovulation rate was determined according to Viñoles *et al*. [29] with minor modifications. The evaluation was performed by observing the collapse of large follicles (≥5 mm) followed by assessing the presence and counting of luteal tissues at the same site 8 days later.

#### Intraovarian blood flow

Color doppler was used to determine the intraovarian perfusion blood flow. Evaluations were performed on the 6^th^ day (Figure-1) using a color Doppler ultrasound scanner equipped with a 7.5 MHz linear transrectal probe (Mindray DP 2200 VET, Mindray Bio-Medical Electronics Co.). The settings of the scanner (Doppler sampling frequency pulse repetition frequency = 1.0 kHz, depth = 6.5 cm, and color gain = 100%) remained constant for the duration of the study. Quantitative analysis of the blood flow area (ImageJ^®^) was used to assess intraovarian blood flow in the Doppler images, as described by Oliveira *et al*. [37], with modifications. Briefly, in the images containing the cross-sectional area of the ovary with the strongest color Doppler signal, the ovary was outlined manually, and the total ovarian area (TA) was recorded. Subsequently, the color doppler area (DA) representing follicular blood flow was computed for each ovary. Finally, the DA percentage (DA/TA × 100) was obtained for each day and each goat.

#### Ovarian artery hemodynamics

The left and right ovarian arteries were localized immediately after the intraovarian blood flow ultrasound evaluation from the 5^th^ day to the 7^th^ day to evaluate the Doppler velocimetric parameters of the ovarian arteries. Blood flow was determined at the most prominent color spot in the ovarian pedicle within 5 mm of the ovarian base. The ovarian artery was identified as a single vessel winding around the ovarian vein. Thus, the ovarian artery blood flow waveforms were obtained by activating the pulsed Doppler function and placing a Doppler gate with a diameter of 2 mm over the colored ovarian artery at this location. The values of end-diastolic velocity (EDV) and peak systolic velocity (PSV) were calculated using the spectral Doppler waveform and estimated by the mean value of the measurements from both ovaries. The pulsatility index (PI) was calculated by subtracting the EDV from the PSV and dividing by the time-averaged (mean) velocity (TAV): PI = (PSV–EDV)/TAV. The angle of insonation between the Doppler ultrasound beam and flow direction in the ovarian artery was 60°.

### Metabolites and glutathione peroxidase (GPx) assays

Blood samples were collected on the 6^th^ day every 6 h (06:00, 12:00, and 18:00) using heparinized vacutainer tubes (Labor import, Wei Hai, China) before feeding in the morning. The samples were centrifuged at 600× *g* for 15 min. The plasma obtained was stored at −20°C for further quantification of metabolites. The plasma concentrations of glucose and cholesterol were determined using an automated biochemical analyzer (Mindray BS 120, Mindray Bio-Medical Electronics Co.) and commercial kits (Bioclin, Quibasa, Minas Gerais, Brazil). The sensitivity of the assay kit was 1.5088 mg/dL for glucose and 1.472 mg/dL for cholesterol. Glutathione Peroxidase was analyzed using a semi-automatic biochemical analyzer (Randox RX Monza TM, Randox Laboratories, Crumlin, UK) and commercial kits (Randox Laboratories) with a sensitivity of 75 U/L.

### Statistical analysis

Statistical analyses were performed using Statistica Software, version v. 13.4.0.14 (TIBCO Software, Inc., Palo Alto, CA, USA). Data were initially verified for mathematical assumptions by Kolmogorov-Smirnov and Bartlett’s tests. When these conditions were not respected, the log10× transformation was applied.

*In vivo* performance, feed intake, follicular dynamics, and metabolite levels were subjected to analysis of variance (ANOVA) using the general linear model (GLM) procedures in a factorial arrangement, where the main effects tested were the group (GM0, GM5, GM10, and GM20), the effect of the interval of assessment used (time), and interaction (group vs. time). Descriptive ultrasonography data regarding ovulatory rate and intraovarian blood flow were analyzed using the GLM procedure, with the group as a factor model. For data on carcass markers and ovarian artery hemodynamics, the GLM procedures for repeated measures of ANOVA with effects tested were the group, the effect of the interval of assessment used (time), and interaction (group vs. time). The recorded anatomical images (1, 2, and 3) were repeated. Data from *in vivo* performance, feed intake, and dynamic follicles were subjected to polynomial contrasts to test the linear and quadratic effects of increasing doses of microalgae. All pairwise comparisons were performed using the Newman-Keuls *post hoc* test, which was applied when ANOVA indicated a significant difference (p < 0.05).

## Results

### *In vivo* performance and feed intake

The ambient temperatures (Min\Max) recorded during the experiment were 28.7 ± 1.7°C and 28.8 ± 1.8°C, respectively. The humidity (Min\Max) was 71.7 ± 7.8% and 72.2 ± 7.5%, respectively. The relative effect of microalgae dose increment on DM intake was significant (Table-2), expressed as g\kg MW (p = 0.0330) and % BW (0.0119). In the comparison between means, the treatment with 20 g of microalgae showed a lower consumption (p < 0.05) than the other groups (Table-2). The live weight of the animals (Table-2) and the thickness of the subcutaneous lumbar fat were similar between the microalgae groups during the experimental period (p = 0.3396). The last parameter showed a significant increase in all groups during the experimental period (time effect, p = 0.0005). A quadratic increase (p = 0.0156) was observed in the microalgae group for renal fat.

**Table-2:** Means and standard errors of dry matter intakes, body weight, subcutaneous lumbar fat thickness, kidney fat thickness, and follicles dynamics in goats fed with increasing dose of green microalga.

Attributes	Groups				p-value				
	
	GMA 0	GMA 5	GMA 10	GMA 20	Groups	Time	G versus T	Linear	Quadratic
Feed Intake									
DMI, g/kg MW	76.0 ± 1.5^a^	71.2 ± 1.0^ab^	74.6 ± 1.5^a^	69.3 ± 2.0^b^	0.0330	0.3710	0.9999	0.6724	0.8166
DMI, % BW	3.1 ± 0.1^a^	2.9 ± 0.04^ab^	3.1 ± 0.1^a^	2.9 ± 0.1^b^	0.0119	0.4615	0.9999	0.7640	0.6696
Body and carcass markers									
Body weight, kg	37.3 ± 1.2	37.4 ± 1.1	36.5 ± 0.9	39.2 ± 0.8	0.3396	0.4986	0.9894	0.3577	0.2062
SLFT, mm	3.9 ± 0.1	4.0 ± 0.1	4.0 ± 0.1	3.9 ± 0.1	0.8833	0.0005	0.3349	0.3635	0.2843
KFT, mm	2.5 ± 0.04^a^	2.3 ± 0.02^b^	2.5 ± 0.1^a^	2.5 ± 0.03^a^	0.0165	0.7857	0.8474	0.0278	0.0156
Follicular dynamics*									
Total follicles, n\ovary	2.3 ± 0.1^a^	2.7 ± 0.1^b^	3.3 ± 0.1^c^	3.2 ± 0.1^c^	<0.001	0.1167	0.9452	<0.001	0.0478
Follicles<3 mm, n\ovary	0.8 ± 0.1^a^	1.1 ± 0.1^a^	1.6 ± 0.1^b^	1.6 ± 0.2^b^	<0.001	0.2512	0.9945	0.0113	0.1663
Follicles≥3 mm, n\ovary	1.5 ± 0.1	1.6 ± 0.1	1.7 ± 0.1	1.6 ± 0.1	0.6927	0.9273	0.9924	0.3758	0.3412
Mean follicle size, mm	3.8 ± 0.1	3.7 ± 0.1	3.5 ± 0.1	3.6 ± 0.1	0.4070	0.3063	0.9955	0.2994	0.4837

DMI=Dry matter intake, MW=Metabolic weight, BW=Body weight, SLFT=Subcutaneous loin fat thickness, KFT=Kidney fat thickness. Time, ANOVA effect for interval of assessment used. ^a,b,c^Columns with different letters between groups (GMA0, GMA5, GMA10, and GMA20), differ (p<0.05). Contrast: Linear=Linear effect of increasing level of microalga; Quadratic=quadratic effect of increasing levels of microalga.

* Follicles traits performed by ultrasonography on the 0^th^ Day to the 4^th^ Day

### Glucose, GPx, and cholesterol levels

**Figure-2:**
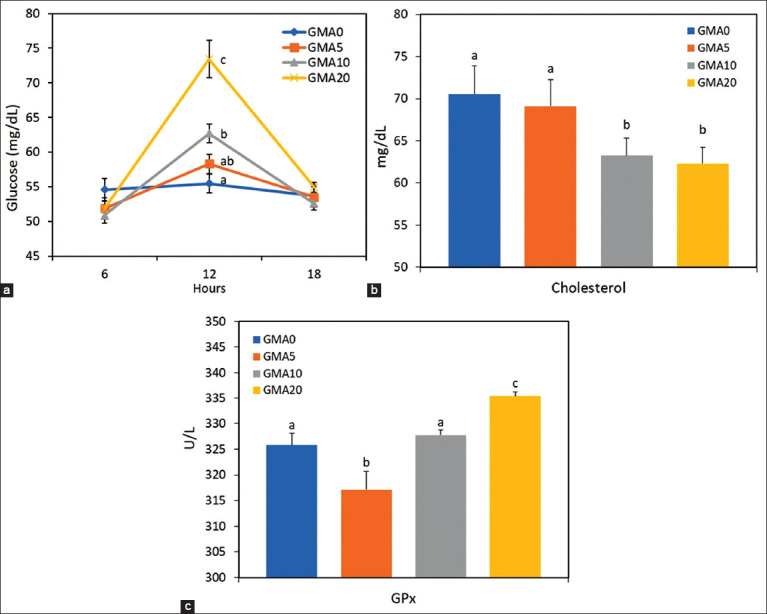
Peripheral glucose (a) measured during sampling every 6 h (06:00 h am, 12:00 h am, 18:00 h pm) on the 6^th^ day. No interactions and effects of the sampling interval were verified in cholesterol and glutathione peroxidase. (b and c) Overall mean group levels. Values are expressed as mean ± standard error of the mean. ^a,b,c^p < 0.05 differences between groups.

Figure-2a illustrates the glucose concentrations recorded during the 24 h period. There was a highly significant interaction between microalgae dose and measurement time (p < 0.001). Glucose levels increased significantly after 12 h as a function of increasing microalgal supply, peaking in the GMA20 group. After 12 h, glucose concentrations decreased and remained at similar values between the treatments. There was no significant effect of the sampling interval or the interaction between the group and time, while the group was significant for cholesterol and GPx. Figures-2b and c show the group means for plasma cholesterol and GPx over the 6^th^ day of the experimental range. The GMA20 group had the highest concentration (p < 0.05) of GPx compared with the other treatments (Figure-2c). Cholesterol levels were significantly reduced (p < 0.05) in the GMA10 and GMA20 groups (Figure-2b).

### Ovarian response and ovarian-intraovarian blood flow

During the experimental period, there was a quadratic increase (p = 0.0478) in the number of total follicles (Table-2) as a function of microalgae dose. At the same interval, the number of follicles <3 mm increased linearly (p = 0.0113) with supplementation. The GMA10 and GMA20 groups recorded the highest values (p < 0.05) between treatments for both parameters. There were no differences in follicular diameter.

**Figure-3:**
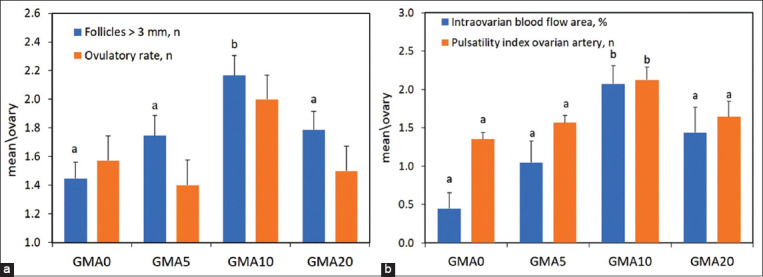
Number of follicles >3 mm counted after the third application of prostaglandin F2 alpha (PGF2α) analog and ovulatory rate (a) determined by ultrasonography after the third application of PGF2α analog. Color Doppler intraovarian area of the ovary and pulsatility index of the ovarian artery recorded at ovulation induction (b). Values are expressed as mean ± standard error of the mean. ^a,b^p < 0.05 differences between groups.

Figure-3a presents the data reactive to the number of follicles with a diameter >3 mm in the period of inducing ovulation after administration of the third dose of prostaglandin in the hormonal protocol. There was a significant increase (p < 0.05) in the follicular class in the GMA10 group. This treatment also resulted in a higher (p < 0.05) area of blood perfusion and PI of the ovarian artery (Figure-3b). The GM10 group exhibited a 25% higher ovulation rate than the other treatments (Figure-3a). However, no statistical differences were observed between the doses of microalgae applied.

## Discussion

The results obtained in this study reveal that oral supplements of green microalgae act efficiently on ovarian follicular development in goats when provided for a short interval. Our evidence points to dosages of 10–20 g of microalgae daily as the best treatment for increasing the number of follicles before ovulation. However, the results after ovulation induction suggest that supplementation with 10 g provides the best ovarian response (increasing the number of follicles >3 mm), accompanied by greater intraovarian blood perfusion.

The inclusion of microalgae in the diet of ruminant animals has been widely explored in tropical regions because of their nutritional potential [16]. However, for use in ruminants, there remains no consensus on dosages and the duration of usage. Therefore, the study enabled us to fill this gap and propose an effective short supplementation protocol to maximize ovarian activity in goats. Notably, the choice of the study dose range was based on the previous studies [21, 22].

In recent years, several authors have focused their attention on short-term food supplementation strategies or specific nutritional interventions to improve the reproductive efficiency of animals, especially in tropical and subtropical areas [38–40]. These approaches may represent a viable solution in tropical regions due to the disadvantage of sustaining feed intake at high temperatures for long periods, where high-energy diet programs with high-quality feed are often competitive with human diets [41]. Short food formulations have advantages regarding management and cost and efficient modulation of ovarian activity in goats [42, 43] and sheep [44, 45].

Follicular growth is dependent on the action of gonadotropic stimulation, controlled by the action of metabolic-hormonal axes [46]. The increase in glucose and reduction in plasma cholesterol observed at the dosages of 10 and 20 g of microalgae suggest that these mechanisms possibly acted in this study. In addition, this greater metabolic availability was associated with an increased and more efficient blood supply at the ovarian level.

In addition to its high production of nutrients, microalgae, particularly *Chlorella*, is characterized by the presence of growth factors [47] and has peptides capable of exerting regulatory activity in the circulatory system. Some studies have identified species such as *Chlorella vulgaris*, *Chlorella sorokiniana*, *Spirulina platensis*, and *Tetradesmus obliquus* are listed among the species that present the IAPG peptide, which demonstrates an angiotensin-converting enzyme inhibitory effect [48].

Changes in metabolites such as cholesterol may promote increased steroidogenesis and an increased ovulation rate. Thus, an increase in glucose availability is related to an increase in circulating Insulin-like growth factor 1 (IGF-1) concentration, which modifies ovarian sensitivity to gonadotropins [49]. The IGF system is involved in follicular proliferation and development [50]. Therefore, the follicular growth results in our study are likely due to IGF type I receptor activation. However, in combination with IGF-1, follicle-stimulating hormone (FSH) indirectly controls follicular development by allowing follicles to access blood glucose, thereby promoting increased steroid production from granulosa cells [51]. An increase in plasma glucose concentration had an inhibitory effect on neuropeptide Y release. Inhibition of neuropeptide Y promotes the release of GnRH from the hypothalamus, which in turn promotes FSH and Luteinizing Hormone pulses [52]. In addition to improving the ovulation rate, increased blood flow allows for a better supply of hormones, nutrients, and oxygen during follicle development, which may be associated with better quality of the ovulated oocyte [53].

In sheep and goats, the development of antral follicles in the ovary is most susceptible to the effects of short diets, which also act in animals with high body conditions accompanied by live weight variations [46]. Our evidence revealed that despite no significant changes in the weight of the animals, there was a quadratic increase in visceral fat as a function of the increase in the oral dose of microalgae. This phenomenon was not unexpected, considering that adult goats in good body condition tend to accumulate energy reserves in adipose tissue in the viscera (fat around kidneys and mesenteric fat) more intensively than other ruminants [54].

Except for the number of small follicles, which increased linearly with the supplement dosage, the results highlighted the general limits of the animal response at the highest oral dosage (20 g). In this group, there was a decrease in food intake and an increase in plasma GPx concentration, which was accompanied by ovulation induction by a reduction in large follicle growth and intraovarian blood perfusion.

Reduced diet intake containing algae or microalgae has already been observed in goats [18]. Moate *et al*. [55] also observed reduced intake in dairy cattle fed algal meal containing 20% DHA. According to Altomonte *et al*. [56], reduced intake may occur due to decreased palatability in addition to decreased fiber digestibility, small particle size that may negatively influence rumen pH, and altered rumen fermentation through PUFA in microalgae, which may have toxic effects on rumen microflora.

The inclusion of fatty acids in the diet can alter the oxidative balance. According to Guerra *et al*. [41], the inclusion of a source of fatty acids in the diet can increase serum glutathione concentrations, reduce lipid peroxidation, and minimize oxidative stress. A fact that may be associated with *C. vulgaris* is being a good source of UFA, glycoproteins, and carotenoids, and containing the growth factor Chlorella, which is a detoxifier and has the tendency to inhibit oxidation [57]. Alves *et al*. [42] found similar results with increased GPx in a lipid diet. Khalil *et al*. [58] demonstrated that high GPx levels promote greater cellular protection against oxidative damage.

There is an extensive list of metabolites and nutrients that directly affect follicular function. The intrafollicular mediators of nutritional influences on folliculogenesis, including glucose, insulin, leptin, growth hormone, and IGF, are the most studied [59]. In this study, a high glucose concentration was observed in the supplemented group, which corroborates the findings of Dos Santos *et al*. [60], who demonstrated that fatty acid supplementation promotes higher serum glucose concentrations in sheep. The previous studies have reported that glucose can induce ovarian development and steroid hormone secretion through the hexosamine pathway, *in vivo* [61]. However, glucose has deleterious effects on ovarian structure and function at higher concentrations because of the inhibition of granulosa cell proliferation and secretion of steroid hormones [62, 63]. According to Matsui and Miyamoto [64], there is a strong relationship between blood flow and follicular growth, indicating that larger follicles have greater detectable blood flow. Thus, impaired follicular growth due to elevated glucose levels may result in reduced blood flow to the ovaries.

## Conclusion

Oral administration of increasing doses of microalgae for a brief period stimulated the growth of the follicular population in tropical goats, reaching maximum values at daily doses of 10 g and 20 g. However, the 10 g supplement proved to be the most effective choice in reproductive terms, considering the higher increase in large follicles and more efficient blood perfusion during ovulation.

## Authors’ Contributions

DR: Supervision, data curation, writing - preparation of the original draft, and acquisition of funds. MRLS, CMC, AJHC, AFB, and ACSS: Sample collection, analysis of samples and results, and drafted the manuscript. MRLS: Figure design. JPMA, CCLF: Preparation of original draft, methodology, interpreted the data and wrote the manuscript. DIAT, ACR: Data analysis, supervised the study, and reviewed the manuscript. All authors have read, reviewed, and approved the final manuscript.
